# Influence of Social Support Network and Perceived Social Support on the Subjective Wellbeing of Mothers of Children With Autism Spectrum Disorder

**DOI:** 10.3389/fpsyg.2022.835110

**Published:** 2022-03-24

**Authors:** Xiao-bin Bi, Hui-zhong He, Hai-ying Lin, Xiao-zhuang Fan

**Affiliations:** ^1^Department of Special Education, Faculty of Education, East China Normal University, Shanghai, China; ^2^Department of Special Education, Faculty of Education, Beijing Normal University, Beijing, China; ^3^Shanghai Rehabilitation Center for Children With Disabilities, Shanghai, China

**Keywords:** autism spectrum disorder, mother, social network, perceived social support, subjective wellbeing

## Abstract

This study explored the relations between the social support network of mothers of children with autism spectrum disorder (ASD), perceived social support, and their subjective wellbeing. The participants were mothers of children with ASD in Shanghai. Their social support network structure was explored *via* the nomination method. Perceived social support was measured using the Revised Social Provisions Scale for Autism (R-SPS-A), and the mothers’ subjective wellbeing was assessed using the Index of Wellbeing, Index of General Affect. A significant correlation was observed between the subjective wellbeing of mothers of children with ASD and perceived social support. Meanwhile, perceived social support was significantly correlated with the effectiveness of overall social support. Finally, perceived social support was also significantly correlated with the network size of social support. Moreover, the effectiveness of social support was significantly associated with the network size of social support and was highly significantly associated with the degree of intimacy of social support. Furthermore, the network size of instrumental support has a significant influence on all perceived social support subdimensions. Overall, social support effectiveness plays an important role in the social support network mechanism on perceived social support and subjective wellbeing in China.

## Introduction

Autism spectrum disorder (ASD) is characterized by early-onset alterations in an individual’s social communication and interactions along with restricted, repetitive patterns of behavior, interests, or activities and sensory abnormalities (American Psychiatric Association, 2013). Around 2% of children worldwide are diagnosed with ASD (Maenner et al., 2020; Zablotsky and Black, 2020); in China, the observed ASD prevalence rate was 0.29% (Zhou et al., 2020). Children with ASD experience significant functional impairment and mental health issues, resulting in a low quality of life for them and their family (DePape and Lindsay, 2015; Lord et al., 2020). Also, parents encounter many difficulties in raising children with ASD (Burrell et al., 2017; Al et al., 2019); they have to deal with their children’s problem behaviors (Scheerer et al., 2021), bear the high financial costs of intervention and rehabilitation (Lavelle et al., 2014; Rogge and Janssen, 2019), cope with parental conflicts (Hartley et al., 2010, 2017), and partake in less leisure and entertainment activities (Walton, 2019). Most importantly, in most families, mothers always shoulder the main parenting responsibility of children with ASD (Estes et al., 2012; DePape and Lindsay, 2015; Papadopoulos, 2021). In addition, parents of children with ASD are always in an extended state of high stress (Hayes and Watson, 2013), which negatively affects their physical, emotional, and functional health (Murphy et al., 2007; Kuru and Piyal, 2018), especially the mothers (Beer et al., 2013; Jones et al., 2013; Hartley et al., 2018). In turn, the poor health of mothers of children with ASD affects both the development of these children and balance within the family system (Karst and Van Hecke, 2012). Many studies have suggested the importance of social support to the physical and mental health of mothers of children with ASD (Lu et al., 2015; Xavier and Wesley, 2018; Zhao et al., 2021); such perceived social support helps alleviate their stress, anxiety, depression, and other negative psychological conditions (Chen S. D. et al., 2021; Koukouriki et al., 2021; Scheerer et al., 2021) and facilitates the enhancement of their parenting competence and the improvement of their wellbeing (Iadarola et al., 2017; Green et al., 2021; Khusaifan and El Keshky, 2021). Meanwhile, the less social support felt by mothers of children with ASD, the more they suffer from psychological problems such as anxiety and depression (Smith et al., 2011; Huang et al., 2019; Bujnowska et al., 2021).

Social support refers to an individual’s perception of the availability of help or support from others in their social network (Berkman et al., 2000; Cohen, 2004). It is often differentiated through three types of resources: instrumental support, which involves providing material aid; informational support, which refers to providing relevant information intended to help the individual to cope with difficulties; and emotional support, which involves empathy, care, and trust and provides opportunities to express one’s emotions (Dour et al., 2014). Social support is generally measured in terms of perceived social support and received social support. While received social support is an index of how often an individual obtains certain supportive behaviors, perceived social support pertains to people’s beliefs about the extent and quality of support available from their relationships and social contacts (Hupcey, 1998; Dour et al., 2014). Measures of perceived social support assess the quality or adequacy of social support from a subjective perspective. Its measurement feasibility makes it the most commonly evaluated social support index (Ibarra-Rovillard and Kuiper, 2011). In addition, compared with other measures, perceived social support is a better predictor of mental health and support utilization (Holt-Lunstad et al., 2010).

Social networks have three dimensions: structure, functions, and dynamics. Structure consists of a set of bonds established among people and among networks. In this dimension, social networks may be interpreted in terms of size; density; composition, including types of bonds; and homogeneity, representing the proportion of bonds. Functions, meanwhile, are defined as the support provided by the network. The dynamics of the networks is carried out by the information movement and redistribution.

A breadth of studies have suggested the important role of social networks in maintaining physical and mental health (Peer and Hillman, 2014; Latkin and Knowlton, 2015; Jones and Storksdieck, 2019; Lin et al., 2019), but scholars have yet to explore such an effect further (Hunter et al., 2019; Chen Y. T. et al., 2021). Originally, the relation between social networks and individuals’ physical and mental health was viewed from two perspectives. One holds that social network directly affects one’s physical and mental health by increasing their social participation and social integration (Pescosolido and Georgianna, 1989). The other believes that social network has an indirect effect on one’s physical and mental health, which means that it influences individuals’ psychological adaptation by shaping their perceived social support, reducing their degree of depression and promoting their subjective wellbeing (Thoits, 2011).

More comprehensive theoretical models, such as relational regulation theory and the convoy model, were used to clarify the mechanism of social networks as it pertains to health. Relational regulation theory views the impact of social support on one’s physical and mental health from the perspective of the interaction between the individual and their social network (Lakey and Orehek, 2011; Rodwell and Munro, 2013; Lakey et al., 2021). This theory states that the interaction between the support provider and the support recipient dictates the role that social support will assume. Social support is shaped by elements of the relationship between the provider and recipient, which means that social support delivered by the same support provider to different recipients will have different effects, while social support delivered by different providers to the same recipient will work differently. Meanwhile, the convoy model examines the longitudinal growth of an individual’s social network from a life development perspective (Antonucci et al., 2014; Pachner et al., 2020) and points out that social support comes from one’s social network, which consists of family, friends, and other social members. An individual’s social network, as a conveyor, benefits their physical and mental health through life and work. Studies on social support and social relationships are witnessing the increasing significance of the interaction process between individuals and their social relations and the structural characteristics of social support networks. Specifically, taking the individual’s social support network as the starting point is important to analyze the relation between their received social support, their subjective social support, and their mental health. A clarified social support mechanism regarding individual mental health helps provide guidance and basis for clinical intervention and social support. Indeed, a few studies have explored these relationships by combining received and perceived social support (Benson, 2012, 2016). Two studies explored the association between characteristics of the social support network, the subjective feeling of social support, and the wellbeing of mothers of children with ASD based on the convoy model. They reported that structural (network size and proportion of emotional support members in social networks) and functional indicators could predict the perceived social support and subjective wellbeing of mothers of children with ASD (Benson, 2012, 2016).

Social support is a variable that is well-known to be influenced by society, economy, and culture. However, studies have not sufficiently clarified whether the social support and subjective wellbeing in the Chinese socioeconomic and cultural context are the same as those in the Western context (Lei and Kantor, 2021). One study focusing on the composition and effectiveness of social support networks for mothers of children with ASD found that different social relationships provided different social support functions and that both the degree of relationship intimacy and frequency of social contact had significantly positive correlations with social support effectiveness (He and Lin, 2013). Meanwhile, the role of social support for mothers of children with ASD in China and the relation between their social support network, their perceived social support, and their subjective wellbeing remain unknown. With the sharp increase in the incidence of ASD, the number of Chinese families dealing with ASD has increased rapidly (Zhou et al., 2020). This raises the urgency of providing effective social support to ASD families, especially mothers, to reduce the pressure of raising children with ASD and improve their wellbeing. Accordingly, it is significant and valuable to study the relation between social support network, perceived social support, and the wellbeing of mothers of ASD children in China.

Based on the existing literature, we will explore the social support network structure and measure the perceived social support and subjective wellbeing of mothers of ASD children in the Chinese cultural context. We will also analyze whether the characteristics of different social support networks influence the different dimensions of subjective social support of mothers of ASD children. Finally, we will examine the hypothesis that social support network indirectly affects the subjective wellbeing of mothers of children with ASD through social support effectiveness.

## Materials and Methods

### Participants

The study was approved by the University Committee on Human Research Protection of East China Normal University. A convenience sample of mothers of ASD children at intervention institutions and auxiliary schools in Shanghai was identified, because convenience sampling is cheap, efficient, and simple to implement (Jager et al., 2017). Participants were enrolled if they met the following inclusion criteria: (1) the participant had at least one child diagnosed with ASD; (2) the participant was willing to attend the study. After informed consent was obtained, participants were visited to perform the assessments face to face and the interviews by phone.

### Measures

At the first stage, the participants filled out a demographic questionnaire, which included items on mother-and-child characteristics, and two assessment tools on the perceived social support and subjective wellbeing of mothers. At the second stage, each participant participated in phone interviews where they discussed issues regarding sources of social support in caring for their children. The structured interview included nine questions: (1) If you need to go out temporarily, who will you ask to help you take care of the children? (2) If you encounter financial difficulties in raising the children, who would you ask for help (including anyone except your spouse)? (3) If you want to take children out, who can you ask to go with you? (4) Who can give you advice on how to care for the children? (5) Who can give you the medical information or advice on the children? (6) Who can give you the information or advice on the education of the children? (7) Who will you talk to when you are in a bad mood for the children? (8) Who will you consult when making decisions on the issues about the children? (9) If you have disputes or conflicts with your spouse (or family) over the children, who will you talk to? Specifically, they identified people who can help them with these problems and explained their relationship intimacy, and contact frequency with these individuals. Finally, the participants evaluated the effectiveness of the social support provided by their nominated persons. We then performed pairwise correlations between characteristics of social support network, perceived social support, and subjective wellbeing and path analysis to explore the impact mechanism of social support network on perceived social support and subjective wellbeing.

Perceived social support was measured using the Revised Social Provisions Scale for Autism (R-SPS-A). The Social Provisions Scale (SPS) consists of six dimensions: attachment, social integration, reassurance of worth, reliable alliance, guidance, and opportunity for nurturance (C E Cutrona and Russell, 1987). Attachment refers to interpersonal relationships that bring people comfort and security. Social integration is the social relationship formed by common interests, which includes one’s sense of belonging in social participation through opportunities to interact and share experiences. Reassurance of worth pertains to other people’s recognition of an individual’s ability to perform their social role. Reliable alliance mainly refers to the availability of other people who can be depended on to provide significant help in difficult times. Guidance is the delivery of advice and support by reliable and authoritative individuals. Opportunity for nurturance pertains to one’s sense of responsibility.

In a study involving 1,792 subjects including students, nurses, and teachers, the Cronbach α value was 0.92, with each subscale’s internal consistency coefficient ranging from 0.65 to 0.76 (Cutrona and Russell, 1987). In addition, reliable alliance, reassurance of worth, social integration, and guidance in the SPS could predict the occurrence of postpartum depression in mothers (Russell et al., 1987). Since the participants of the present study were mothers of children with ASD, we adapted the original SPS scale to make it more consistent with our research purpose. First, the SPS scale was translated into Chinese with the assistance of English-major graduate students. Second, the items for “reassurance of worth,” “reliable alliance,” and “guidance” in the revised scale were adapted to the questions applicable to mothers of ASD children. For example, “There are people I can depend on to help me if I really need it” was revised to “When I need to make decisions related to children with ASD, there is someone I can discuss with,” and several corresponding items were added. Third, the mothers of children with ASD as well as experts were consulted regarding the original revised questionnaire, which was repeatedly improved to ensure that the meaning of the translated and revised questionnaire was clear. Finally, the modified scale was used for item analysis, factor analysis, and structural validity verification. The final version of R-SPS-A was used to test the internal heterogeneity of both the overall and subdimension questionnaires.

Subjective wellbeing was measured using the Index of Wellbeing, Index of General Affect (Campbell, 1976). This index measures individual happiness and includes two parts: The Index of General Affect consists of eight items describing the connotation of affect from different levels, while the Index of Wellbeing had only one item. Ratings for each item in the overall index ranged from 1 to 7 points. The total score of the Index of Wellbeing, Index of General Affect was calculated by adding the average scores of its two parts (weight 1.1), with scores ranging from 2.1 to 14.7.

The purpose of the nomination method was to measure the social support network of mothers of children with ASD (He and Lin, 2013). For each social support item, the participants nominated individuals whom they can depend on. We divided the interview outline for social support network into three dimensions—instrumental support, information support, and emotional support—each with three items (He and Lin, 2013). Instrumental support includes temporary care of children, financial assistance, and taking children out together. Meanwhile, information support includes advice on taking care of children with ASD, medical and educational information, and guidance pertaining to children with ASD. Finally, emotional support includes discussing decisions related to ASD children and conflicts between individuals and their families. In the interview, the participants nominated a maximum of five people for each item, and their nominated individuals formed their social support network. The participants then provided information about each member in their social support network, including gender, degree of relationship intimacy, and contact frequency.

The social support network was measured by considering network size, relationship strength in the network, and social support effectiveness. Network size refers to the number of social relationships that constitute the social support network and is categorized into three: network size of instrumental support, network size of information support, and network size of emotional support. The total social support network was formed by the participants’ nominations for nine items, while the subnetwork was formed by the participants’ nominations for the subdimensions.

Meanwhile, relationship strength was measured using contact frequency and degree of intimacy. Contact frequency was scored using a 6-point scale: “almost daily” (6), “almost weekly” (5), “monthly” (4), “several times a year” (3), “once or twice a year” (2), and “less” (1). Degree of intimacy used a 10-point scoring system: complete strangers (0 points) and general level of intimacy (5); the higher the score, the more intimate the relationship.

Finally, social support effectiveness was measured by scoring the effectiveness of each social support item as follows: “highly effective” (4), “effective” (3), “general” (2), and “no effect” (1).

## Results

### Descriptive Statistics

A total of 174 original R-SPS-A and 110 Index of Wellbeing, Index of General Affect were collected from 182 participant. Among the 182 participants, 104 participants who completed both original R-SPS-A and 110 Index of Wellbeing, Index of General Affect were invited to attend the interview. Among the 104 participants, 64 participants completed the interview. Finally, the final sample analyzed in the study was constituted by the 64 participants who completed the two questionnaires (original R-SPS-A and Index of Wellbeing, Index of General Affect) and the interview. The children of the 64 participants had an average age of 102.48 months (SD = 39.904 months) and were predominantly male (85.9%). Regarding the participants’ age, 42.2% (*n* = 76) were below 35 years old, 40.6% (*n* = 26) were between 36 and 40 years old, and 17.2% (*n* = 11) were above 41 years old. More than half (57.8%) did not hold bachelor’s degrees, and 32.8% did not perform any type of job. Table 1 shows the detailed demographic information of both the mothers and their children.

**TABLE 1:** Basic characteristics of participants and their children.

Item	Number	Percent (%)	Total
Age (mothers)			64
<35	27	42.2	
36–40	26	40.6	
>41	11	17.2	
Education level (mothers)			64
Senior high school and below	23	35.9	
Junior college	14	21.9	
Bachelor degree or above	27	42.2	
Working status (mothers)			64
Full-time/part-time	43	67.2	
Unemployed and other else	21	32.8	
Gender (children)			64
male	55	85.9	
female	9	14.1	
School (children)			64
Preschool	19	29.7	
Primary school	36	56.3	
Junior middle school	9	14.1	

Perceived social support was measured using the final version of the R-SPS-A. According to the descriptive information of the R-SPS-A, 5 corresponding items were added to the original 24 SPS items, resulting in 29 total items under 6 dimensions for the original version of the R-SPS-A, which was sent to the target participants. The participants returned a total of 174 accomplished copies of the original R-SPS-A. These valid samples then underwent item analysis and reliability and validity tests. The average scores for each item in the high group (i.e., samples at the top 27%) and the low group (samples at the bottom 27%) underwent t-test. The difference in the average scores for each item in the high and low groups was either significant or extremely significant; hence, all the 29 items in the original R-SPS-A were retained. To verify the structural validity of the original R-SPS-A, the 29 were analyzed *via* factor analysis, which included the Kaiser–Meyer–Olkin value (KMO) and Bartlett’s test of sphericity. The KMO value was 0.826, while the alpha value for Bartlett’s test of sphericity was 718.014, indicating significance (*p* = 0.000) and data suitability for factor analysis. A confirmatory factor analysis was then conducted, starting with principal component analysis for standardized factor loadings using varimax rotation in SPSS to explore the items’ underlying constructs. After repeatedly deleting and retaining items and conducting factor analysis, 15 items were finally retained. A total of four factors (guidance, social integration and reliable alliance, attachment, and reassurance of worth) were synthesized, with a total interpretation rate of 57.93%. Cronbach’s alpha coefficients were calculated to assess the internal consistency reliability of the items in the final version of the R-SPS-A. The Cronbach’s alpha coefficients were 0.850 for the R-SPS-A and 0.635–0.762 for the four subscales, indicating good internal consistency. The overall score for perceived social support was 2.90 ± 0.512 as measured by the R-SPS-A. Scores for guidance, social integration and reliable alliance, attachment, and reassurance of worth as measured by R-SPS-A were 2.83 ± 0.722, 2.87 ± 0.622, 3.11 ± 0.715, and 2.74 ± 0.593, respectively. This means the participants were satisfied with perceived social support, especially in the attachment dimension, which had the highest score. Table 2 shows detailed statistics.

**TABLE 2:** Perceived social support and subjective wellbeing.

Variable	M	SD
Perceptive social support	2.90	0.512
Guidance	2.83	0.722
Social integration and reliable alliance	2.87	0.622
Attachment	3.11	0.715
Reassurance of worth	2.74	0.593
Subjective wellbeing	8.77	1.960

Subjective wellbeing was measured *via* the Index of Wellbeing, Index of General Affect. A total of 110 participants returned the accomplished instruments, which were tested for internal consistency, resulting in a Cronbach’s alpha coefficient of 0.874. The average score for the General Affect Index (average value of seven items) was highly significantly correlated with that of the Index of Wellbeing (with one item) (*p* < 0.01), with a correlation coefficient of 0.707. Therefore, the instrument was suitable for measuring the participants’ subjective wellbeing. The score for subjective wellbeing was 8.77 ± 1.960 as measured by the Index of Wellbeing, Index of General Affect, indicating that mothers of children with ASD had a medium level of subjective wellbeing. Table 2 shows detailed information.

The nomination method was used to measure social support network. The mothers of children with ASD had an overall network size of 2–11 with an average size of 6.84. Regarding relationship strength within the participants’ social network, the overall average score for contact frequency was 5.18 ± 0.554, between “almost weekly” and “almost daily.” Meanwhile, the overall average score for degree of intimacy was 7.30 ± 1.170. The degree of intimacy of the emotional support network was the highest at 7.96 ± 1.351 points, while the degree of intimacy of the information support network was 6.40 ± 2.084 points, the lowest among the three types. The effectiveness of social support was 2.97 ± 0.453, which was close to “effective.” Table 3 displays detailed statistics.

**TABLE 3:** Description of social support network characteristics.

Variable	*M*	SD
Network size of social support	6.84	2.018
Network size of instrumental support	3.94	1.781
Network size of information support	2.88	1.638
Network size of emotional support	3.30	1.797
Degree of intimacy of social support	7.30	1.170
Degree of intimacy of instrumental support	7.72	1.251
Degree of intimacy of information support	6.40	2.084
Degree of intimacy of emotional support	7.96	1.351
Contact frequency of social support	5.18	0.554
Contact frequency of instrumental support	5.42	0.648
Contact frequency of information support	4.52	1.381
Contact frequency of emotional support	5.41	0.601
Effectiveness of social support	2.97	0.453
Effectiveness of instrumental support	3.06	0.624
Effectiveness of information support	2.79	0.784
Effectiveness of emotional support	2.88	0.632

### Correlation Analysis and Path Analysis

This study performed a correlation analysis of the characteristics of social support network, effectiveness of social support, perceived social support, and subjective wellbeing of mothers of children with ASD. A significant correlation was observed between the subjective wellbeing of mothers of children with ASD and perceived social support (*p* < 0.05). Perceived social support was significantly correlated with effectiveness of overall social support (*p* < 0.05) and network size of social support (*p* < 0.05). In addition, effectiveness of social support had a significant association with network size of social support (*p* < 0.05) and a highly significant association with degree of intimacy of social support (*p* < 0.01). Table 4 shows detailed information.

**TABLE 4:** Correlation between social support network, effectiveness of social support, perceived social support, and subjective wellbeing.

	Network size of social support	Degree of intimacy of social support	Contact frequency of social support	Effectiveness of social support	Perceived social support	Subjective wellbeing
Network size of social support	1	–0.065	0.003	0.257*	0.301*	0.032
Degree of intimacy of social support		1	0.059	0.520**	0.104	0.118
Contact frequency of social support			1	0.136	–0.040	0.087
Effectiveness of social support				1	0.298*	0.164
Perceived social support					1	0.356*

*p < 0.05 and **p < 0.01.

Correlations were found between the characteristics of the social support network, effectiveness of social support, perceived social support, and subjective wellbeing of mothers of children with ASD, but the interaction and influence of these variables on each other remain unknown. Hence, we analyzed such interactions via path analysis.

**FIGURE 1:**
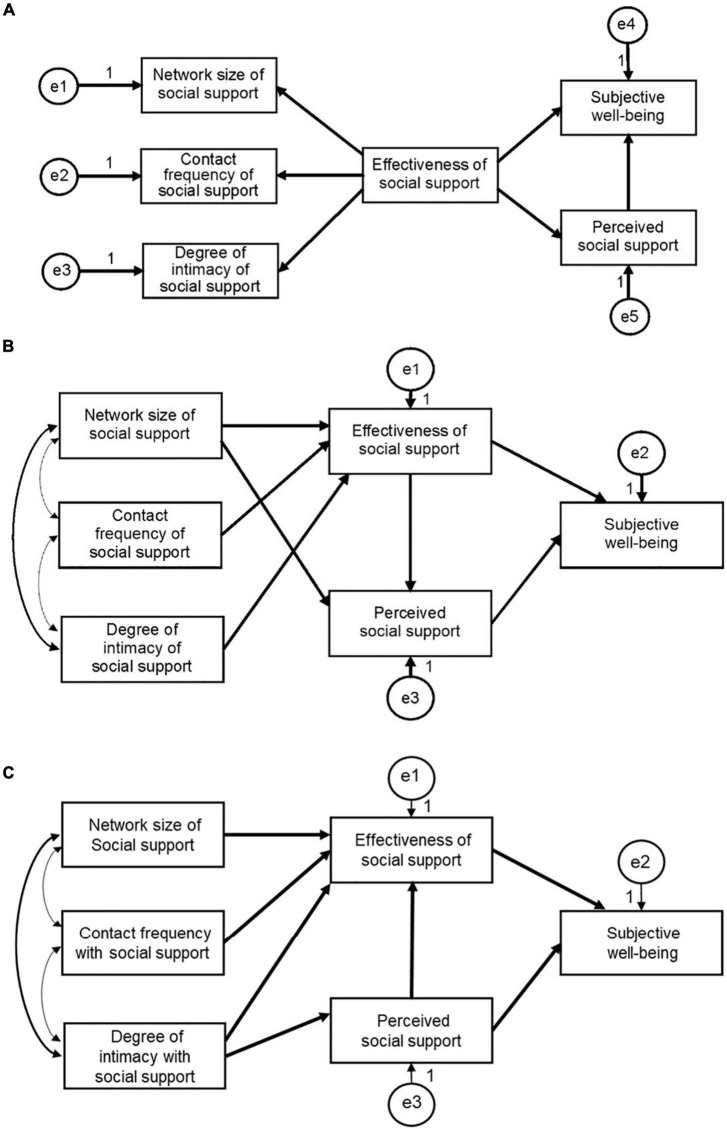
Hypothetical models of the effect of social support network on perceived social support and subjective wellbeing. **(A)** Hypothetical model 1. **(B)** Hypothetical model 2. **(C)** Hypothetical model 3.

From the correlation analysis results, the possible path models of the influence of social support network on perceived social support and wellbeing were proposed, as shown in Figure 1. In hypothetical model 1, the effectiveness of social support affects perceived social support and subjective wellbeing. Also, the individual’s experience of social support effectiveness affects the extent to which an individual seeks such support; that is, social support effectiveness affects the social support network size, degree of intimacy, and contact frequency, as shown in Figure 1A. Meanwhile, in hypothetical model 2, social support network characteristics (network size, degree of intimacy, and contact frequency) influence social support effectiveness and thus affect perceived social support and then subjective wellbeing, as shown in Figure 1B. Finally, hypothetical model 3 assumes that perceived social support affects the perception of social support effectiveness based on hypothetical model 2, as shown in Figure 1C.

Confirmatory path analysis of the three hypothetical models was conducted using AMOS 7.0. Table 5 shows the models’ fit indexes; the closer the X2/df is to 0, the better the model–data fit. For RMSEA, which refers to the square root of the average square error, the closer the value is to 0, the better the model fit. Usually, an RMSEA < 0.1 and even < 0.05 indicate good model fit. For GFI, the closer the value is to 1, the better the model fit; generally, GFI < 0.9 should be guaranteed for the model fit. NFI refers to the benchmark fitness index, whose value is between 0 and 1; the higher the NFI value, the better model–data fit. A comparative fitness index equal to 1 indicates that the data fits the model completely. Incremental fitness index (IFI) values are between 0 and 1; an IFI equal to 1 shows that the data fits the model completely. A comparison of the three models shows that model 2 has the best data fit.

**TABLE 5:** Fit index of the path analysis models for total social support network.

Model	X^2^	Df	X^2^/df	RMSEA	GFI	NFI	CFI	IFI
Model 1	9.843	9	1.094	0.039	0.949	0.801	0.976	0.979
Model 2	1.610	6	0.268	0.000	0.992	0.967	1.000	1.101
Model 3	7.560	5	1.512	0.090	0.963	0.847	0.926	0.942

**FIGURE 2:**
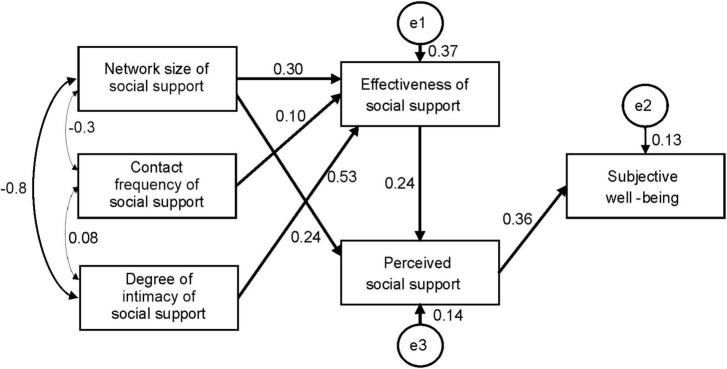
Path analysis of the influence pattern of social support network on perceived social support and wellbeing.

Figure 2 shows the path analysis results for the influence of social support network on the perceived social support and wellbeing based on model 2. The path coefficients of network size of social support to social support effectiveness and degree of intimacy to social support effectiveness were 0.30 (*p* < 0.01) and 0.53 (*p* < 0.001), respectively. Both the path coefficients of network size of social support and effectiveness of social support to perceived social support were 0.24 (*p* < 0.05). Also, the path coefficient of perceived social support to subjective wellbeing was 0.36 (*p* < 0.01). All these suggest that the degree of intimacy of social support affected perceived social support through its influence on the effectiveness of social support and finally the mother’s subjective wellbeing. Meanwhile, the network size of social support affected mothers’ subjective wellbeing through its influence on the effectiveness of social support and perceived social support.

Correlation analysis was performed between the characteristics of the instrumental, information, and emotional support network of mothers of children with ASD and the subdimensions of perceived social support. Network size of instrumental support was significantly correlated with guidance, social integration and reliable alliance, and reassurance of worth (*p* < 0.05) and highly significantly correlated with attachment (*p* < 0.01). A significant correlation was observed between degree of intimacy of information support and reassurance of worth (*p* < 0.05), and contact frequency of information support was also significantly correlated with reassurance of worth (*p* < 0.05). Table 6 shows detailed figures.

**TABLE 6:** Correlation analysis between the characteristics of social support network sub-dimensions and perceived social support sub-dimensions.

	Guidance	Social integration and reliable alliance	Attachment	Reassurance of worth
Network size of social support				
Instrumental support	0.334*	0.276*	0.345**	0.260*
Information support	0.183	0.019	0.162	0.195
Emotional support	0.146	0.102	0.309*	0.191
Degree of intimacy of social support				
Instrumental support	0.026	0.107	0.106	0.137
Information support	0.186	0.093	0.000	0.294*
Emotional support	–0.093	0.053	–0.023	0.049
Contact frequency of social support				
Instrumental support	–0.057	–0.065	–0.054	–0.027
Information support	0.212	0.045	–0.005	0.296*
Emotional support	0.072	0.126	0.128	–0.116

*p < 0.05 and **p < 0.01.

**FIGURE 3:**
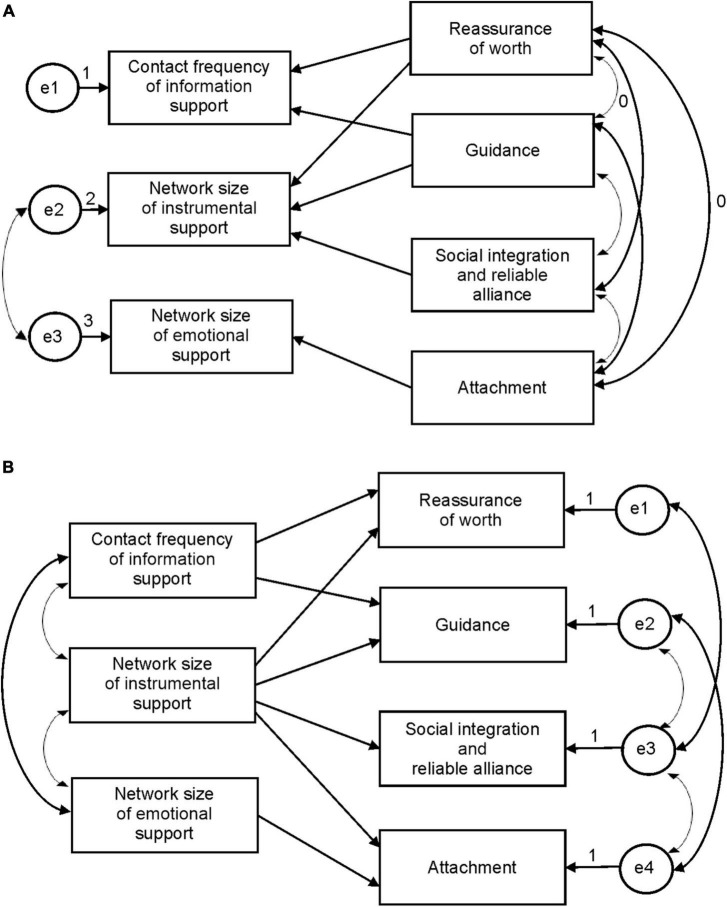
Hypothetical models of path analysis based on social support network and perceived social support. **(A)** Hypothetical model 1. **(B)** Hypothetical model 2.

**FIGURE 4:**
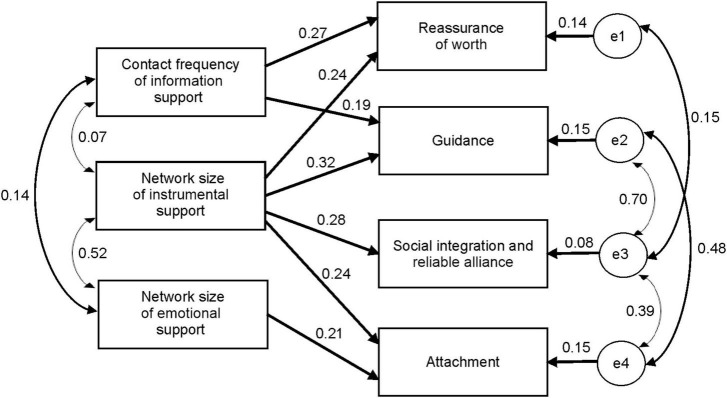
Path analysis of the influence pattern of social support network on perceived social support.

Correlation analysis further explored the relation between the subdimensions of social support network and subdimensions of perceived social support. Path analysis of the influence of social support network variables on the perceived social support was conducted. Two hypothetical models were then proposed as shown in Figure 3. Hypothetical model 1, shown in Figure 3A, indicates that subdimensions of perceived social support affect social support network characteristics, while hypothetical model 2, shown in Figure 3B, indicates that social support network characteristics affect individuals’ perceived social support. Table 7 shows the model fit indicators after the two models were verified. Each fit index showed a larger error between model 1 and the data and a better degree of fit between model 2 and the data. Therefore, the results support hypothetical model 2; that is, social support network characteristics affect an individual’s perceived social support. Figure 4 shows the specific path analysis diagram. The path analysis of social support network and perceived social support showed significant path coefficients, but the path coefficients of network size of emotional support were not significant for attachment (*p* = 0.076 and *p* = 0.073, respectively). The contact frequency of information support affects two subdimensions of perceived social support: reassurance of worth and guidance. The network size of instrumental support significantly influences all perceived social support subdimensions. Finally, the network size of emotional support affects one subdimension of perceived social support: attachment.

**TABLE 7:** Fit indexes of hypothetical models of path analysis based on social support network and perceived social support.

Model	X^2^	df	X^2^/df	RMSEA	GFI	NFI	CFI	IFI
Model 1	16.039.	10	1.604	0.098	0.933	0.870	0.941	0.947
Model 2	3.166	7	0.869	0.000	0.986	0.974	1.000	1.033

## Discussion

The present study explored the social support network of mothers of children with ASD and introduced the variable of social support effectiveness to analyze the impact of social support network characteristics on mothers’ perceived social support and subjective wellbeing. Significant correlations were observed between the subjective wellbeing of mothers of children with ASD and perceived social support and between perceived social support and social support effectiveness. In addition, perceived social support was also significantly correlated with the network size of social support. The path analysis results suggest that the network size of social support and the degree of intimacy of social support further affected perceived social support and subjective wellbeing by influencing social support effectiveness. Also, the network size of social support also had an impact on perceived social support, which further affected subjective wellbeing. The current results indicate that social support network indirectly affected the subjective wellbeing of mothers of children with ASD through the effectiveness of social support and perceived social support. To some extent, the present study validated the indirect mechanism of social networks on the physical and mental health of mothers of children with ASD (Benson, 2012), which was consistent with the view of the convoy model; that is, perceived social support had a greater impact on individual mental health than objective and actual social support (Pachner et al., 2020). Moreover, the present study found that the degree of social support intimacy of mothers of children with ASD influenced social support effectiveness, thus affecting perceived social support and subjective wellbeing. This result verified relationship regulation theory to a certain degree; that is, social support takes its role from the interaction between support providers and support recipients and is shaped by the characteristics of their relationship (Lakey and Orehek, 2011).

According to the path analysis of the relation between social support network characteristics and perceived social support of mothers of children with ASD, different functions of social support network characteristics influence the different dimensions of their perceived social support. Among these dimensions, the contact frequency of information support affects guidance and reassurance of worth. Information support refers to the delivery of child care advice, medical information, and educational information. The information support network mainly consists of teachers and other parents of children with ASD (He and Lin, 2013). The path analysis results show that the more frequent the engagement of mothers of children with ASD with individuals in the information support network, the more they feel supported. In addition, mothers of children with ASD felt that they were recognized for their ability to take care of their children. The network size of instrumental support influenced all perceived social support dimensions: guidance, social integration and reliable alliance, attachment, and reassurance of worth. Instrumental support mainly refers to the temporary care of children with ASD, the delivery of financial support, and taking the children out, which are conducted by family members and relatives. The network size of instrumental support is crucial to mothers of children with ASD. Because of the serious behavioral problems of children with ASD, parents of these children have less leisure time and social activities, which aggravates the parents’ psychological pressure (Heiman and Berger, 2008). Instrumental support can alleviate the burden of mothers who take care of children with ASD. The network size of instrumental support may also positively affect all perceived social support dimensions, while the network size of emotional support affects the attachment dimension of the perceived social support. Emotional social support refers to the unburdening and discussions of conflicts with family members, mainly provided by spouses and friends. If mothers have more friends who can provide emotional support, they would feel more comfortable in their interpersonal relationships. Notably, the contact frequency of information support affected mothers’ subjective feelings of social support, while all instrumental support and emotional support variables affected their perceived social support. This difference may be because the information support network mainly consists of unrelated teachers and other parents, and the contact frequency of informational support manifests the amount and degree of intimacy of social support. However, instrumental support and emotional support are mainly provided by relatives and close friends with high contact frequency and high degree of intimacy. Therefore, the network size, not the degree of intimacy and contact frequency of instrumental support and emotional support, affected mothers’ perceived social support.

Social support effectiveness plays an important role in social support network mechanism on perceived social support and subjective wellbeing. Provided by members of the social support network, social support effectiveness affects mothers’ perceived social support, thus shaping their subjective wellbeing. The social network and social support of mothers of children with ASD influence their wellbeing by affecting their subjective cognition, such as social support effectiveness, perceived social support, and so on. When providing social support for mothers of children with ASD, besides helping them build a relatively perfect social network, one must also pay attention to their perceived social support and whether such support meets their needs. The contact frequency of information support shapes mothers’ perceived social support, thus influencing their subjective wellbeing. This necessitates an expansion of the source of information support for mothers of children with ASD. When delivering informational support for mothers of children with ASD, professionals must also maintain a certain contact frequency and focus on the continuity of support supply. Other parents of children with ASD are important sources of informational support. Therefore, mothers of children with ASD should participate in more parent associations and related parenting training to benefit from other parents of children with ASD. The network size of instrumental support significantly affects their perceived social support, thus shaping their subjective wellbeing. Family members and other relatives are the main providers of instrumental support, and mothers should actively seek their assistance to alleviate their burden in raising and taking care of their children with ASD. The findings in the present study have implications for the provision of social support for mothers of children with ASD. Teachers should try to help the mothers to better educate and care for ASD children, as well as to provide them how to find medical help for their children. Furthermore, it is important to improve the education and maintenance skills of mothers of children with ASD, which will greatly promote the improvement of mothers’ wellbeing. In addition, it is helpful for mothers of children with ASD to live in a large family and make contact frequently with family members. The husband and relatives should provide substantial support to the mothers of ASD children by taking more responsibilities in the family. From the perspective of providing social support to mothers of children with ASD, it is essential that providers should strengthen the contact frequency with mothers, for the goal of social support could not be achieved in a short time. Also, it is useful to provide different kinds of social support, due to the changes of the needs of mothers of ASD children along with the growth of children.

There are several limitations in the present study. First, the sample only partially represents these mothers who would like to make use of the intervention, training and educational services for ASD children. It is difficult to rule out sampling biases due to the basis of the convenience sampling in the study. Thus, the present findings may not be generalized to all mothers with ASD children. Secondly, child factors, especially intellectual functioning should have been considered as relevant dependent variables or covariates in this study, for how to deal with ASD children’s complications, such as intellectual disabilities, was an important reason why mothers of ASD children seek social support. Different social support needs of mothers are accompanied with different kinds of complications of ASD children. Thirdly, the present study was a cross-sectional study which examined social support and wellbeing of mothers of ASD children at one time point, but social support and wellbeing should be considered as a dynamic process that changes over time.

Future research is needed to furtherly clarify the influence of social network and social support on the wellbeing of mothers of ASD children. Future researches need to adopt more effective sampling methods to recruit more representative samples, so that the generalizability of study findings could be improved. Furthermore, the influence of social network and social support on the individual’s physical and mental health is complex. Individual and situational factors will also affect the mechanism of social network and social support on one’s physical and mental health (Scheid and Brown, 2010). However, the correlation and path analyses in the present study excluded the personal characteristics of children with ASD and their mothers. Therefore, future studies must consider more factors to better clarify the mechanism in the impact of one’s social support network on their physical and mental health. In addition, social networks and relationships bring not only social support to mothers of children with ASD but also conflicts, contradictions, and other social tensions, which may negatively affect their physical and mental health (Lakey and Orehek, 2011). Hence, future research should examine the negative effects of social network relationships as well. Finally, from the conceptual framework of the convoy model, individual social network and social support is a dynamic process. Therefore, researches which could generate longitudinal data to establish causal connections among the characteristics of social network, social support and wellbeing of mothers of ASD children is needed.

## Data Availability Statement

The raw data supporting the conclusions of this article will be made available by the authors, without undue reservation.

## Ethics Statement

The studies involving human participants were reviewed and approved by the University Committee on Human Research Protection of East China Normal University. The patients/participants provided their written informed consent to participate in this study.

## Author Contributions

X-BB and H-YL designed the study, conducted the research, analyzed the data, wrote the manuscript, conceived the idea of the study, collected the data, and interpreted the results. H-ZH conceived the project and supervised the study and provided a theory guide and supervised the study. H-ZH and X-ZF coordinated the project and edited the manuscript. All authors discussed the results, approved the manuscript, and read and approved the manuscript.

## Conflict of Interest

The authors declare that the research was conducted in the absence of any commercial or financial relationships that could be construed as a potential conflict of interest.

## Publisher’s Note

All claims expressed in this article are solely those of the authors and do not necessarily represent those of their affiliated organizations, or those of the publisher, the editors and the reviewers. Any product that may be evaluated in this article, or claim that may be made by its manufacturer, is not guaranteed or endorsed by the publisher.
